# Peripheral white blood cell patterns in children with hydrocephalus as a response to ventriculo-peritoneal shunt infection

**DOI:** 10.1371/journal.pone.0308131

**Published:** 2024-08-09

**Authors:** Bartosz Polis, Krzysztof Zeman, Krzysztof Zakrzewski, Artur Fabijan, Emilia Nowosławska

**Affiliations:** 1 Department of Neurosurgery, Polish Mother’s Memorial Hospital- Research Institute in Lodz, Lodz, Poland; 2 Department of Pediatrics, Immunology and Nephrology, Polish Mother’s Memorial Hospital- Research Institute in Lodz, Lodz, Poland; University of Michigan Medical School, UNITED STATES OF AMERICA

## Abstract

Shunt infection is one of the most common complications of conventional hydrocephalus treatment. The route of invasion of a pathogen can modify the immune response of the CNS. The aim of the study is to analyze the immune response to shunt infection caused by S. epidermidis in children with hydrocephalus. The immune response to the pathogen will be analyzed on the basis of, inter alia, simple laboratory test results, such as changes in the pattern of white blood cells, including neutrophils, monocytes, and lymphocytes. The entire study analyzes changes in general parameters of the cerebrospinal fluid (pleocytosis, protein level, glucose level) and in levels of selected interleukins (IL-6, CXCL8 / IL-8, CCL3 / MIP-1a) in the cerebrospinal fluid. The clinical material analyzed in the study was collected in 2010–2014. The study group consisted of 30 patients, who were admitted to the hospital due to their first-ever episode of valve dysfunction caused by S. epidermidis infection. The control group consisted of 30 children who also suffered from congenital hydrocephalus but had not been operated on before. The most pronounced response to CSF infection in the study group was a significant increase in the counts of all investigated WBC lines in the samples collected immediately after the patients’ admission to the ward. The earliest aberration of the CSF was a significant increase in protein level. An infection of a ventriculoperitoneal shunt caused by S. epidermidis evokes a very early peripheral blood response. In children affected by a ventriculoperitoneal valve infection, the humoral immune response detected in the cerebrospinal fluid precedes the increase in the level of pleocytosis. The highest level of cytokines in the cerebrospinal fluid is achieved when the pathogens are cleared. Phagocytes, and, in particular, monocytes, play an important role in the normalization of the cerebrospinal fluid parameters after the elimination of S. epidermidis. The local immune response of the central nervous system plays an important role in extinguishment of the inflammatory process.

## Introduction

Shunt infection is one of the most common complications of conventional hydrocephalus treatment. Bacterial contamination can occur not only during surgical treatment, but also during a puncture of the atrium of the ventriculo-peritoneal valve system performed in order to collect a sample of the cerebrospinal fluid (CSF). Therefore, each decision to take a CSF sample should be well-founded. It should be made after analysis of clinical symptoms and basic laboratory blood tests. White blood cell pattern results would be particularly useful for this purpose. The central nervous system is an immunologically privileged site. The immune response within it is suppressed but inducible [1–4]. The factors responsible for these unique abilities of the central nervous system are the blood-brain barrier (BBB), the blood-cerebrospinal fluid barrier (BCB) and lack of expression of the histocompatibility system [5]. The immune response can also be modified by intraventricular administration of antibiotics [1, 5–8]. It has been noted that their intrathecal administration may increase the number of white blood cells [6]. The path of bacterial invasion determines which type of meningitis develops. The meningitis which occurs after ventriculoperitoneal valve implantation may not be typical. Pneumococcal meningitis usually occurs as a consequence of penetration of the bacteria through the mucosa of the respiratory tract, whereas patients suffering from ventriculoperitoneal shunt infection are at risk of introducing bacterial material directly into CSF. Thus, the route of infection does not require the blood- cerebrospinal fluid barrier (BCB) to be crossed. The route of invasion of a pathogen can modify the immune response of the central nervous system (CNS). This may explain why ventriculoperitoneal shunt infections relatively rarely lead to severe encephalitis and meningitis [4, 9, 10]. The aetiology of ventriculoperitoneal valve infections differs significantly from the aetiology of the meningitis caused by pathogens which invade the central nervous system through the conventional route. The most common bacterial pathogens causing encephalomyelitis are *Streptococcus pneumoniae* (*S*. *pneumoniae)* (58% of cases) and *Neisseria meningitidis* (*N*. *meningitidis*) (19% of cases) [4, 10–13]. The most common organism responsible for shunt infections is *Staphylococcus epidermidis (S*. *epidermidis)* (32–70% of cases) [14, 15]. Another one is *Staphylococcus aureus* (*S*. *aureus)* (12–48%). *S*. *pneumoniae* ranks third and it is responsible for 6–10% of the infections [14, 15]. Due to the high frequency of infections caused by *S*. *pneumoniae*, the immune response to it is relatively well understood. Knowledge of the immune response in ventriculo-peritoneal valve infection caused by *S*. *epidermidis* is very often based on animal models [14, 15]. This issue is worth exploring for several important reasons. The problem is significant, as it is considered that the implantation of an intraventricular catheter is the most common procedure in neurosurgery. The immune response can be weakened by formation of biofilm on the surface of the intraventricular catheter, which prevents elimination of the pathogen [16]. An intraventricular shunt infection affects local immune response of the CNS. The inflammatory process initially excludes interactions of the pathogen with antigen presenting cells (APCs), such as peripheral macrophages or dendritic cells. This affects many processes; for example, it results in a delay in interleukin secretion, as APCs are a source of peripheral interleukins synthesised outside the CNS [10]. The aim of the study is to analyze the white blood cell pattern in children with hydrocephalus, which expresses the immune response to shunt infection caused by *S*.*epidermidis*. The immune response to the pathogen will be analyzed on the basis of, inter alia, simple laboratory test results, such as changes in the pattern of white blood cells, including neutrophils, monocytes, and lymphocytes. The entire study analyzes changes in general parameters of CSF (pleocytosis, protein level, glucose level) and in levels of selected interleukins and chemokines (IL-6, CXCL8 / IL-8, CCL3 / MIP-1a) in CSF.

## Materials and method

The clinical material analyzed in the study was collected from November 10, 2010, to March 15, 2014. All guardians of the patients recruited for the study signed an informed consent form for participation in the study, prior to inclusion, while also providing consent for the use of biological material for immunological research. The experiment in no way affected the diagnostic and therapeutic procedures. The study and control groups were children hospitalized at the Research Institute of the Polish Mother’s Health Centre, Lodz, Poland. All the children met the established inclusion criteria. The study group consisted of 30 patients. All of them were admitted to the hospital due to their first-ever episode of valve dysfunction caused by S. epidermidis infection. All the children in the study group suffered from congenital communicating hydrocephalus. The study selectively included children diagnosed with congenital hydrocephalus who had undergone a shunt revision due to their first CSF infection. This criterion was chosen to focus on a specific subset of hydrocephalus cases with comparable clinical profiles. The age limit for participants was set at under 18 years, ensuring the study focused exclusively on a pediatric cohort. The general condition at the time of birth of children included in the study group, expressed by the Apgar scale, should be not less than 10 points. A crucial prerequisite for inclusion in the study was obtaining informed consent from the patient’s guardians. This consent was vital not only from a legal standpoint but also to ensure that the guardians were fully aware of and agreeable to the study’s aims and procedures.

On the other hand, the study set clear boundaries regarding exclusion criteria to maintain the integrity and focus of the research. Any cases of hydrocephalus that did not align with the congenital origin. This exclusion extended to any patients who had undergone shunt revision for reasons other than infection or were undergoing subsequent shunt revisions. Moreover, individuals over the age of 18 were not considered to maintain the pediatric scope of the study. To exclude sources of induction of inflammatory processes other than increased CNS intracranial pressure, none of the children with complex events during the perinatal period were included in the study group. Therefore, children with intraventricular hemorrhage or perinatal ischemic injury were excluded from the study. Another critical aspect of the exclusion criteria was the lack of informed consent from guardians. This included scenarios where guardians either did not sign the consent form or did not fully comprehend the information regarding the study’s purpose and conditions, which could potentially compromise the ethical conduct and results of the research.

These criteria were meticulously designed to ensure a homogenous study group, thereby enhancing the validity and reliability of the findings in the context of pediatric congenital hydrocephalus and its treatment.

The study group consisted of 16 boys and 14 girls. The mean age of the patients was 8.4 months (± 5.8 months). The control group consisted of children who also suffered from congenital hydrocephalus but had not been operated on before. These children, as those in the study group, suffered from congenital communicating hydrocephalus without concomitant diseases. The control group included thirty children who were treated during the study period. The group consisted of 17 boys and 13 girls. The mean age of the patients was 1.2 months (± 0.3 months). The study involved analyses of the patients’ CSF and blood samples. The following blood parameters were tested: white blood cell count (WBC), neutrophil count (NEUTRO), neutrophil percentage (NEUTRO%), lymphocyte count (LYMPH), lymphocyte percentage (LYMPH%), monocyte count (MONO) and monocyte percentage% (MONO%). The analysis of CSF samples included determination of general parameters such as: protein level, pleocytosis, and glucose level. Additionally, tests were carried out to measure levels of selected interleukins and chemokines: IL-6, CXCL8 / IL8, CCL3 / MIP-1a in the collected CSF samples. In the study group, blood and CSF samples for analysis were collected three times. The first measurement of the analyzed parameters was performed when ventriculoperitoneal shunt infection was diagnosed. Another measurement took place when the first sterile CSF sample was obtained. The final measurement was taken at the time of the valve reimplantation. In the control group, the blood and CSF samples were taken at the time of valve implantation. The treatment carried out in the study group was standardized. When microbiological testing revealed an infection, the infected ventriculoperitoneal shunt was replaced with a Rickham reservoir. Each patient received targeted intraventricular therapy. *S epidermidis* was susceptible to vancomycin in all patients in the study group. The patients received 5 mg of vancomycin diluted in 1 ml of physiological saline once a day. The intraventricular therapy was continued until the valve was implanted. The described antibiotic treatment took an average of 20.2 days (± 7.9 days) until the first sterile sample was obtained. Another two sterile specimens were required for the valve implantation. To sum up, the valve reimplantation was performed not only after the CSF had become sterile, but also after obtaining the appropriate parameters that enabled the valve mechanism to function properly. The mean time from the initiation of the antibiotic therapy to the valve reimplantation was 23.8 days (± 5.27 days). The parameters analyzed in the study group were measured in the blood and CSF samples of the control group only once. All the CSF samples were stored at -80°C. Prior to testing, the samples were warmed to -20°C and then to room temperature. The cerebrospinal fluid samples were centrifuged at 2000 rpm for 10 min to remove their cellular components. 2.0 ml of supernatant was withdrawn. ELISA was performed to obtain the quantifications. Pre-assembled standard kits (R&D Systems, Inc.; 614 McKinley Place NE; Minneapolis, MN, 55413, USA (Enzyme Immunoassay)) were used. The absorbance readings of the tested samples were performed using a DS2 microplate reader, Dynex Technologies, Inc., USA. The assay was performed using cell culture supernatants. This method was found to be more sensitive and reliable than the determination of the parameters in blood serum. Saline was used as a negative control. The absorbance values for each sample were read at 450 nm. The concentration values of the tested samples were calculated automatically from the standard curve. The sensitivity of the measurements preformed in the provided material, that is, the minimum detectable concentration, was 0.31–0.20 ng / ml. In the study group, the parameters of the cerebrospinal fluid, such as levels of CXCL8 / IL-8, IL-6, CCL3 / MIP-1a, pleocytosis, glucose and protein, obtained in the three measurements were compared using analysis of variance. Additionally, simultaneously with the CSF analysis, the peripheral blood count was checked. Bonferroni’s post hoc analysis was used to detect significant differences between the three measurements. The above parameters obtained in each of the three measurements in the study group were compared with the corresponding parameters in the control group using the Friedman test (Wilcoxon post hoc test with Bonferroni correction for multiple comparisons). To assess normality of the distribution the Kolmogorov-Smirnov test with the Lilliefors correction was used. Homogeneity of variance was examined with the Mauchly test. For non-homogeneous variances the Greenhouse-Geisser correction was applied. The level of statistical significance was set at p < 0.05. SPSS 17.0 software was used for the entire statistical analysis.

## Results

The immune response to *S*. *epidermidis* ventriculoperitoneal shunt infection, expressed as count and percentage of particular types of white blood cells in the peripheral blood, was examined in three measurements in the study group and compared with the response in the control group of patients (see Table 1).

**Table 1:** The count and percentage of particular types of white blood cells, in the peripheral blood in each of the measurements planned for the study. The ANOVA test revealed significant correlations between the measurement results both in the control group and in the study group.

Type of WBC	Measurement I	Measurement II	Measurement III	Control group	Significant differences
WBC (mean cc/μl, st. dev)	16.49(±1.08)	10.96(±1.12)	9.58(±1.20)	8.20(±2.35)	I>II>III>C
Neutrophiles (mean cc/μl, st.dev)	5.70 (±0.47)	3.49 (±0.61)	2.75 (±0.32)	2.26 (±1.58)	I>II>III = C
Neutrophiles% (mean%, st.dev)	37.35 (±2.51)	33.07 (±5.12)	32.02 (±2.06)	43.36 (±7.79)	C>I>II = III
Monocytes(mean cc/μl, st. dev)	0.89 (±0.09)	0.67 (±0.12)	0.63 (±0.09)	0.28 (±0.09)	I>II = III>C
Monocytes% (mean%, st.dev)	5.85 (±0.63)	6.27 (±0.86)	7.34 (±0.72)	6.57 (±2.47)	III>I = II = C
Lymphocytes(mean cc/μl, st. dev)	7.53 (±0.49)	3.67 (±0.59)	4.67 (±0.45)	2.02 (±0.55)	I>III>II>C
Lymphocytes %(mean%, st.dev)	49.37 (±2.30)	34.93 (±5.57)	54.56 (±2.18)	45.50 (±8.27)	III>I>C>II

I- the first measurement, II -the second measurement, III-the third measurement, C- control group.

The results of the analysis of variance performed on the study group showed that there were statistically significant differences between the white blood cell counts (WBC) in each of the measurements (see Table 1): *F*(2, 58) = 352.18; *p* < 0.001. The highest WBC count was detected in the first measurement. It was higher than in the second measurement (*p* < 0.001). The count in the second measurement was higher than in the third measurement (*p* < 0.001). Using the Mann-Whitney U test, it was found that leukocytosis in the control group was lower than in all three measurements in the study group: in the first measurement (*p* <0.001), in the second measurement (p <0.001) and in the third measurement (*p* = 0.009). The analysis of variance showed statistically significant differences in the count of neutrophils in respective measurements (see Table 1): *F*(2, 58) = 286.12; *p* < 0.001. The highest count was found in the first measurement. It was significantly higher than the second measurement (*p* < 0.001). The third measurement revealed the lowest count. It was much lower than that in the second measurement (*p* < 0.001). In the Mann-Whitney U test the control group showed a significantly lower count of neutrophils compared to the counts in first measurement (p < 0.001) and in the second measurement (*p* < 0.001). In the third measurement there was no significant difference between the counts of neutrophils in the control group and in the study group.

The analysis of variance also showed statistically significant differences in the percentage of neutrophils between individual measurements (Table1):*F*(2, 58) = 17.98; *p* < 0.001. The highest percentage of neutrophils was found in the first measurement. It was significantly higher than in the second (*p* = 0.001) and third (*p* < 0.001) measurement. There was no statistically significant difference between the percentage of neutrophils in the second and third measurements (*p* = 0.971). The Mann- Whitney U test showed that in the control group the percentage of neutrophils was significantly higher than in all the measurements in the study group (p < 0.001).

The analysis of variance revealed significant differences in the counts of monocytes between the individual measurements (see Table 1): *F*(2, 58) = 63.14; *p* < 0.001. In the study group the highest count of monocytes was detected in the first measurement (*p* < 0.001). There was no statistically significant difference between the count of monocytes in the second and third measurements (*p* = 0.385). According to the Mann-Whitney U test the count of monocytes was significantly lower in the control group than the study group (*p* < 0.001).

The analysis of variance showed statistically significant differences in the percentage of monocytes between individual measurements (see Table 1): *F*(2, 58) = 30.70; *p* < 0.001. The highest percentage of monocytes was detected in the third measurement (*p* < 0.001). There was no statistically significant difference in the percentage of monocytes between the first measurement and the second measurement (*p* = 0.177). According to the MannWhitney U test, no statistically significant difference in the percentage of monocytes was found between the results of the control group and the results of individual measurements in the study group: in the first (*p* = 0.129), in the second (*p* = 0.533), in the third (*p* = 0.105).

The analysis of variance showed significant differences in the count of lymphocytes between individual measurements (see Table 1): *F*(2, 58) = 410.16; *p* < 0.001. The highest count of lymphocytes was obtained in the first measurement (*p* < 0.001). The lowest count in the study group was recorded in the second measurement (*p* < 0.001). The count of lymphocytes in the third measurement was significantly lower than in the first measurement (*p* < 0.001) but higher than in the second measurement (*p* < 0.001). According to the Mann-Whitney U test, the count of lymphocytes in the control group was significantly lower than in the study group (*p* < 0.001).

The analysis of variance revealed a significant difference between the percentages of lymphocytes in individual measurements (see Table 1): *F*(2, 58) = 231.04; *p* < 0.001. To our surprise, the highest percentage of lymphocytes was found in the third measurement (*p* < 0.001). The lowest percentage was found in the second measurement (*p* < 0.001). According to the Mann-Whitney U test, the percentage of lymphocytes in the control group was higher than in the second measurement in the study group (*p* < 0.001). The remaining measurements in the study group showed a higher percentage of lymphocytes than in the control group (*p* < 0.001).

Another group of obtained clinical data included general parameters of the cerebrospinal fluid and levels of selected interleukins and chemokines in it.

On the basis of the analysis of variance, it was found that there was a statistically significant difference between the levels of protein in the cerebrospinal fluid in the individual measurements (see Table 2): Chi^2^(2) = 60.00; *p* < 0.001. Overall, the highest level was found in the first measurement (*p* < 0.001). In the second measurement it was lower than in the first one (*p* < 0.001). Correspondingly, in the third measurement it was lower than in the second (*p* < 0.001). In the control group, the level of CSF protein was lower than the levels in each measurement in the test group. This was shown by the MannWhitney U test. It was found that the average level of protein in the cerebrospinal fluid in the control group was lower than in the first measurement (*p* < 0.001), in the second (*p* < 0.001) and in the third (*p* = 0.013).

**Table 2:** The left column contains the median and mean values of the assessed general parameters of the cerebrospinal fluid (protein, pleocytosis, glucose) obtained in three measurements in the study group and in one measurement in the control group. The right column presents levels of selected interleukins and chemokines in the study group and the control group. The equations describe statistically significant relationships between the individual measurements in the study group and in the control group (I- the first measurement, II -the second measurement, III-the third measurement, C- control group).

CSF general parameters				Significant differences	Selected interleukins				Significant differences
Protein	Median(mg%)	Statistical mean value (mg%)	Standard deviation mg%	I>II>III>C	IL-6	Median (ng/ml)	Statistical mean value (ng/ml)	Standard deviation ng/ml	II>I> III = C
I	1219.50	1291.63	497.80		I	320.39	393.98	296.92	
II	159.00	179.83	83.12		II	555.05	8107.09	21809.43	
III	26.00	35.30	25.20		III	50.60	101.14	131.36	
C	13.50	17.47	9.24		C	46.35	48.41	11.81	
Pleocytosis	Median (cc/ μl)	Statistical mean value (cc/ μl)	Standard deviation (cc/ μl)	II>I = III = C	CXCL8/IL-8	Median(ng/ml)	Statistical mean value(ng/ml),	Standard deviation ng/ml	C = II>I = III
I	6.50	7.90	7.80		I	991.30	1156.54	555.76	
II	37.00	106.03	145.21		II	1576.35	5258.00	16012.20	
III	5.00	13.10	19.53		III	1328.30	1295.94	395.97	
C	6.00	7.83	6.81		C	1648.40	1704.56	190.74	
CSF glucose	Median(mg%)	Statistical mean value(mg%),	Standard deviation mg%	C>III>I = II	CCL3/MIP-1a	Median(ng/ml)	Statistical mean value(ng/ml),	Standard deviation ng/ml	II>C>III>I
I	35.00	37.83	12.94		I	1.65	7.95	11.40	
II	38.00	38.07	14.65		II	27.28	370.02	1792.01	
III	54.50	89.10	207.43		III	15.69	15.69	13.01	
C	59.50	58.10	10.21		C	17.84	21.30	8.75	

I-first measurement, II-second measurement, III-third measurement, C-control group.

The analysis of variance showed a significant difference in the mean pleocytosis levels in the CSF between the individual measurements (see Table 2): Chi^2^(2) = 12.29; *p* = 0.002. The highest level of cerebrospinal fluid pleocytosis was obtained in the second measurement. It was higher than in the first measurement (*p* < 0.001) and in the third one (*p* = 0.002). There was no statistically significant difference in this parameter between the first and third measurements (*p* > 0.999). On the basis of the Mann-Whitney U test, the mean cerebrospinal fluid pleocytosis level in the second measurement in the study group was significantly higher than in the control group (p = 0.001). The remaining measurements in the study group did not differ significantly from the measurement in the control group (first measurement ‐ *p* = 0.953, third measurement ‐ *p* = 0.976).

The analysis of variance showed statistically significant differences in the mean levels of glucose in the cerebrospinal fluid between the individual measurements in the study group (see Table 2): Chi^2^(2) = 17.36; *p* < 0.001. The third measurement showed the highest level of this parameter. It was significantly higher than in the first measurement (*p* = 0.003) and in the second (*p* = 0.004). However, there was no statistically significant difference between the first and the second measurements (*p* > 0.999). The Mann-Whitney U test showed that in the control group the mean glucose level was significantly higher than the levels obtained in each measurement in the study group (first measurement ‐ p < 0.001, second measurement ‐ *p* < 0.001, third measurement ‐ *p* = 0.027).

The level of IL6 differed significantly between the individual measurements (see Table 2) (Based on the ANOVA test chi2 (2) = 20.87; *p* < 0.001. IL6). It peaked in the second measurement (taken from the first sterile sample). It was statistically higher than in the first measurement (first test indicating CSF infection) (*p* = 0.009). It was also higher than in the third measurement (the test without bacterial contamination and with a decrease in CSF pleocytosis and protein levels, which allowed the ventriculo-peritoneal valve reimplantation) (*p* < 0.001). On the basis of the Mann-Whitney U test, the median of the IL-6 level in the control group was lower than in the first measurement (*p* < 0.001). It was also lower than in the second measurement (*p* < 0.001), which was expected. There was no significant difference between the control group and the third measurement (*p* = 0.865).

The levels of CXCL8 / IL-8 also differed significantly between the individual measurements (see Table 2). (Based on ANOVA chi2 (2) = 18.20; *p* < 0.001). It was particularly surprising that, on the basis of Mann-Whitney U test, the median of CXCL8 / IL-8 level in the control group was higher than in the first measurement (*p* < 0.001) and in the third measurement (*p* < 0.001). Moreover, there was no statistically significant difference between the second measurement and the control group (*p* = 0.408). In the study group, the highest level of CXCL8 / IL-8 was obtained in the second measurement (it was higher than both in the first measurement, *p* < 0.001and in the third, *p* <0.01).

The levels of CCL3 / MIP-1a differed significantly between the individual measurements (see Table 2) (Based on ANOVA chi2 (2) = 32.47; *p* < 0.001). On the basis of the Mann-Whitney U test, significant differences in the levels of CCL3 / MIP-1a were also revealed between the control group and the individual measurements in the study group. In the control group, the CCL3 / MIP-1a level was higher than both in the first measurement (*p* < 0.001) and in the third one (p = 0.013). However, it was lower than in the second measurement (*p* = 0.009). In the study group the highest level was detected in the second measurement. It was higher than both in the first measurement (*p* < 0.001) and in the third one III (*p* = 0.002). Interestingly, in the third measurement it was also higher than in the first (*p* = 0.003).

The relationship between the pattern of peripheral white blood cells and the individual parameters tested in the cerebrospinal fluid was also assessed using Spearman’s rho correlation coefficient. The obtained results were limited only to statistically significant correlations (*p* < 0.05) (see Table 3). The first measurement showed a statistically significant positive correlation between the count of neutrophils in the peripheral blood and the level of pleocytosis in the cerebrospinal fluid. The second measurement showed a positive correlation between the count of monocytes in the peripheral blood and the level of IL-6. Another two statistically significant relationships were revealed in the second measurement. There were statistically significant negative correlations between the count of lymphocytes and the level of CSF protein, and between the percentage of lymphocytes and the level of CSF protein. In the third measurement, two significant relationships were found between peripheral white blood cell pattern and the parameters of the cerebrospinal fluid. A positive correlation was found between the count of lymphocytes and the level of protein. A negative correlation occurred between the count of neutrophils and the level of glucose in the cerebrospinal fluid.

**Table 3:** Statistically significant relationship between the measured CSF parameters and the peripheral white blood cell pattern, based on Spearman’s rho coefficient.

IL6	WBC	NEUTR	NEUTR%	LYMPH	LYMPH%	MONO	MONO%
Measurement II	0.12	-0.16	-0.19	-0.24	-0.20	0.40*	0.40*
Protein	WBC	NEUTR	NEUTR%	LYMPH	LYMPH%	MONO	MONO%
Measurement II	0.09	0.14	0.12	-0.48*	-0.49*	0.31	0.31
Measurement III	0.25	0.29	0.04	0.36*	-0.05	0.23	0.05
Pleocytosis	WBC	NEUTR	NEUTR%	LYMPH	LYMPH%	MONO	MONO%
Measurement I	0.24	0.40*	0.31	-0.01	-0.35	0.03	-0.11
CSF glucose	WBC	NEUTR	NEUTR%	LYMPH	LYMPH%	MONO	MONO%
Measurement III	-0.10	-0.38*	-0.29	-0.21	0.32	-0.13	0.05

WBC- white blood cell count, NEUTR- Neutrophiles, NEUTRO%-neutrophil percentage, LYMPH-lymphocyte count, LYMPH%-lymphocyte percentage, MONO-monocyte count, MONO%-monocyte percentage%.

*- statistically significant.

Another correlation analysis was carried out in the study group and in the control group. The data were limited only to statistically significant and clinically important correlations expressed as Pearson’s Correlation Coefficient (r) (p < 0.05) (see Table 4). In the first measurement, two statistically significant correlations between the counts of particular types of peripheral white blood cells were found. Negative correlations were found between the counts of neutrophils and lymphocytes, as well as between the counts of neutrophils and monocytes (see Table 4). Three positive correlations were found in the third measurement. There was a positive correlation between the counts of neutrophils and monocytes in the peripheral blood. A similar relationship existed between the counts of neutrophils and lymphocytes. Additionally, there was a positive correlation between the counts of monocytes and lymphocytes in the peripheral blood. Negative correlations were found between the percentage of lymphocytes and the count of neutrophils and also between the percentage of lymphocytes and percentage of neutrophils. In the control group negative correlations existed between the percentage of neutrophils and the percentage of lymphocytes and also between the percentage of neutrophils and the count of lymphocytes. A negative correlation also existed between the percentage of lymphocytes and the count of monocytes. Another negative correlation was detected between the count of lymphocytes and the percentage of monocytes.

**Table 4:** Statistically significant relationship between particular types of peripheral white blood cells in the individual measurements in the study group and in the control group, expressed as Pearson’s Correlation Coefficient (r).

Measurement I	NEUTR	NEUTR%	LYMPH	LYMPH%	MONO	MONO%
NEUTR		0.85*	-0.15	-0.78*	-0.20	-0.46*
NEUTR%			-0.62*	-0.92*	-0.37*	-0.40*
LYMPH				0.70*	0.20	-0.09
Measurement III	NEUTR	NEUTR%	LYMPH	LYMPH%	MONO	MONO%
NEUTR		0.62*	0.56*	-0.56*	0.47*	-0.11
NEUTR%			-0.28	-0.89*	-0.11	-0.25
LYMPH				0.33	0.59*	-0.01
Control group	NEUTR	NEUTR%	LYMPH	LYMPH%	MONO	MONO%
NEUTR%			-0.44*	-0.95*	0.17	-0.01
LYMPH				0.57*	-0.13	-0.46*
LYMPH%					-0.42*	-0.29

WBC- white blood cell count, NEUTR- Neutrophiles, NEUTRO%-neutrophil percentage, LYMPH-lymphocyte count, LYMPH%-lymphocyte percentage, MONO-monocyte count, MONO%-monocyte percentage

*- statistically significant

The analysis of correlations between the parameters of the cerebrospinal fluid and the levels of cytokin and chemokines in it was also performed using the Spearman’s correlation coefficient (rho) (see Table 5). Discussion of the obtained data is limited to only the clinically important and statistically significant relationships. In the first measurement, a statistically significant positive correlation was found between the levels of IL-6 and pleocytosis. On the other hand, the correlation between the levels of IL-6 and glucose was negative. The diverse functions of IL-6 in the immune response were also manifested by significant negative correlations of its level with the levels of CXCL8 / IL-8 and CCL3 / MIP-1a. These interleukins and chemokines are chemo-attractants especially for neutrophils, which are crucial in the initial phase of the pathogen invasion. This is shown by a statistically significant positive correlation between the levels of these interleukins and chemokines and the level of glucose. The onset of the inflammatory process is manifested by a positive correlation between the levels of pleocytosis and protein in the cerebrospinal fluid. In the second measurement, the development of the cellular immune response is illustrated by several statistically significant positive correlations between the levels of CXCL8 / IL-8 and pleocytosis, as well as between the levels of CCL3 / MIP-1a and pleocytosis. The important role of IL-6 in the humoral response was illustrated by a positive correlation between its level and the level of protein. On the other hand, in the second measurement there was a shift in the supremacy of immunological response from the cellular to the humoral, which was manifested by a negative correlation between the levels of protein and glucose.

**Table 5:** Analysis of statistically significant relationships between general parameters of the cerebrospinal fluid (glucose, protein, pleocytosis) and the levels of selected interleukins. The table shows statistically significant relationships expressed as the Spearman’s correlation coefficient (rho) (The adopted level of significance, p < 0.05).

Measurement I	IL-6	CCL3/ MIP-1a	Cytosis	Protein	Glucose
CXCL8/ IL-8	-0.38*	0.76*	-0.01	-0.01	0.56*
IL-6		-0.56*	0.52*	0.02	-0.48*
CCL3/ MIP-1a			-0.04	0.21	0.83*
Cytosis				0.44*	-0.06
Measurement II	IL-6	CCL3/ MIP-1a	Cytosis	Protein	Glucose
CXCL8/ IL-8	0.59*	0.70*	0.52*	0.01	0.15
IL-6		0.52*	0.23	0.41*	-0.32
CCL3/ MIP-1a			0.63*	-0.03	-0.04
Cytosis				-0.15	0.05
Protein					-0.55*
Measurement III	IL-6	CCL3/ MIP-1a	Cytosis	Protein	Glucose
CXCL8/ IL-8	0.47*	0.75*	0.22	0.16	-0.44*
IL-6		0.49*	0.52*	0.13	-0.12
CCL3/ MIP-1a			0.33	0.13	-0.44*
Cytosis				0.60*	-0.30
Protein					-0.46*

*- statistically significant

In the third measurement, there were signs of exhaustion of the inflammatory process. This was shown by a negative correlation between the levels of main chemo-attractants cytokines for monocytes and neutrophils and the level of glucose in the cerebrospinal fluid. The residual immune response was also illustrated by a negative correlation between the levels of protein and glucose. The normalization of the parameters of the cerebrospinal fluid in the third measurement allowed an implantation of the ventriculoperitoneal shunt, despite statistically significant positive correlations between the level of IL-6 interleukin and the levels of major chemoattractants cytokines such as CXCL8 / IL-8 and CCL3 / MIP-1a as well as between the levels of IL-6 interleukin and pleocytosis. This may mean that the immune response in the cerebrospinal fluid is not stimulated by the pathogen. CXCL8 / IL8 and CCL3 / MIP-1a had a statistically significant negative effect on the glucose level in the cerebrospinal fluid. It may also explain the decrease in the levels of chemo-attractants due to the removal of pathogens from the cerebrospinal fluid. A statistically significant positive correlation between the levels of protein and pleocytosis may also be an indicator of pathogen clearance.

## Discussion

The clinical material analyzed in the study was collected in 2010–2014. Demographic changes in the country affect the profile of children hospitalized at the Research Institute of the Polish Mother’s Health Center in Łódź. Currently, it would no longer be possible to collect such a homogeneous group of children after valve implantation. Moreover, a decrease in the number of bacterial infections of implanted valves has been observed over the last decade. Thanks to continuous improvement of surgical safety protocols, especially those aimed at eliminating bacterial contamination of implants, such complications occur less frequently. In the presented material, the first measurement illustrates the status of the initial stage of *S*. *epidermidis* infection, before the initiation of treatment. Exposure to the pathogen introduced into the ventricular system first of all leads to induction of the immune response. Due to the specific route of entry, the induction of the immune response depends mainly on the innate local immune system, in which microglia prevail. Understanding of the innate response of the central nervous system to a pathogen invasion has been developed on the basis of experimental animal models and analogies to well-known specific types of bacterial infection. The immune response of the CNS to *S*.*pneumoniae* invasion is relatively well known. The knowledge of the inflammatory process in the CNS in response to *Streptococcus* infection has been discussed in numerous academic publications [4, 10–13]. The most typical route of its invasion into a human organism is the mucosa of the upper respiratory tract. The infection of the human organism by this route triggers both the systemic and the local immune response. At the very onset of the inflammatory process peripheral APCs, such as dendritic cells, monocytes, and macrophages become activated [10, 17]. Immunoglobulin A also takes part in the local immune response to penetration of the mucose membrane by the microorganisms [10, 17]. On the basis of *S*. *pneumoniae* encephalitis, a model of crossing of the blood-brain barrier (BBB) by microorganisms was constructed. The blood-brain barrier consists of tight capillary connections. In order to disrupt the BBB *S*. *pneumoniae*, particularly its transparent form (more resistant to penetration of the BBB than the opaque one), binds with phosphorylcholine to the platelet activating factor (PAF) present in the epithelial wall [10, 11]. The process enables pneumococcal transcytosis across the epithelium. Another route of penetration of the BBB by pneumococci is the binding of their surface protein C (PspC) to laminin (a protein that combines with proteoglycans of the basal membrane, which also contains collagen I). This mechanism enables paracytosis of the pathogen, which is its penetration among the cells of the microvascular epithelium. Another important way of crossing the BBB is passing through the choroid plexus by inducing local epithelial necrosis. [10]. Then, the subsequent colonization path can be divided into several stages. After the pathogen invades the cerebrospinal fluid, it may multiply in the subarachnoid space. It is also possible that, having caused damage to the epithelium, the pathogen can enter the bloodstream. Thus, both the bloodstream and CSF may be pathways for the spread of the pathogen [4, 9, 10]. *S epidermidis* and *Staphylococcus aureus* (*S*. *aureus*), which are most often responsible for ventriculoperitoneal shunt infections, usually use a different route of invasion than *S*. *pneumoniae*. They do not need to transgress the BBB or BCB. The bacterial strains get directly into the cerebrospinal fluid in the intraventricular space [5, 14–16]. In the central nervous system the role of its local immune system is performed by microglia, astrocytes, dendritic cells and macrophages, which are located in the perivascular space and in the meninges [2, 3, 10, 18]. Brain-resident immune cells such as microglia enter the CNS at an early embryonic stage [1, 12]. These cell lines are activated either directly by the pathogen itself or by cytokines [2, 12]. They recognize the pathogen and initiate a local immune response [12]. They are responsible for recognition of pathogen-associated molecular patterns (PAMP) and, to a lesser extent, for the recognition of damage-associated molecular patterns (DAMP) [2, 3, 9, 10, 19]. There are many ways to recognize PAMP in the CNS and signal danger to its local immune system [2, 3, 10, 19]. One of them is stimulation of Toll-like receptors [4, 9]. They can be detected on both nervous and non-nervous structures. These receptors are present not only in astrocytes, oligodendrocytes, and even the neurons themselves, but also in dendritic cells and macrophages in the cerebrospinal fluid. Toll-like receptors may be located both intracellularly and on the cell surface [2, 4, 9, 20]. They form hetero- and homodimer structures and have the ability to get activated through binding to pathogen-associated molecular patterns [4, 9]. Three types of Toll-like receptors (TLR2, TLR4, TLR9) have been identified as the ones that are involved in *S*. *pneumoniae* meningitis [4, 9, 10, 18, 21]. TLR2 and TLR4 are found on the cell surface. TLR9, which is present in the endosomal space, is able to recognize bacterial RNA [4, 9, 18]. In addition to stimulation of toll-like receptors, another method of signalling the presence of a pathogen is induction of the nucleotide-binding oligomerization domain (NOD) protein, which forms an intracellular NOD-like receptor. Activation of the receptors recognizing the pathogen initiates transcription of cytokines and chemokines in the central nervous system. Cytokines contribute to an increase in the permeability of BBB. They can stimulate immune response cells to produce chemokines. Chemokines cause an influx of inflammatory cells from the peripheral blood [2, 4, 9, 20]. Both signalling pathways lead to activation of the serine-threonine protein kinase 2 (NF-κB). This process is of key importance in the innate inflammatory process [9, 10, 12, 18]. NF-κB is essential for production of cytokines and chemokines [8, 10]. The third type of activation of the pathogen recognition receptors involves cleavage of protointerleukin to its active form by inflammasomes. Examples of lymphokines produced from protocytokines are IL-1β and IL-18. The last way of intracellular pathogen recognition involves the SiGNR1 (specific intracellular adhesion molecule-grabbing nonintegrin receptor-1). This process takes place in the microglia. The polysaccharide wall of a pathogen constitutes a molecular pattern which is recognized by this receptor [10]. It is possible that the SiGNR1 receptors may be also present on astrocytes. They are responsible for the improvement of phagocytosis and adhesion as well as for the activation of production of substances participating in the immune response [2]. Once a pathogen-related molecular pattern is recognized, it takes several hours to produce cytokines, including IL-6, which is the subject of this study. This cytokin is responsible for the increase in the permeability of the BBB and activation of cytokine production by APCs, macrophages and natural killers [2, 4, 9, 10, 21, 22]. It is produced not only by microglia but also by other cells located in the central nervous system, such as perivascular and meningeal macrophages, astroglia, and vascular endothelial cells [10, 12]. In an early response, which occurs within 6–24 hours, the active substances are proinflammatory cytokines such as TNFα, INF α/β, IL-1, IL-6,IL-12 [2, 4, 9, 10, 12, 19, 21, 22]. In the course of meningitis the level of cytokines in CSF is clearly higher than in the plasma. The interleukins analyzed in the study (IL-6, CXCL8 / IL-8, CCL3 / MIP-1a) play an important role in the immune response [4, 9, 10, 20, 22]. In the first measurement the level of IL-6 in the cerebrospinal fluid, although much higher than in the control group, did not reach its maximum value, which was obtained in the second measurement(see Table 2). This can be explained by the fact that the cells residing in the CNS which are involved in the immune response are not the only ones that produce IL6. The peripheral blood cells, stimulated by cytokines, may also be engaged in the production of IL6. Macrophages, monocytes, and leukocytes also secrete IL-6. So the increase in IL-6 level in the cerebrospinal fluid could also be explained by its entry into the cerebrospinal fluid through the disrupted BBB [12]. The cells mentioned above may cross the BBB by transcytosis and paracytosis [23]. CXCL8/IL-8 plays an important role in leukocyte recruitment, similar to CCL3 / MIP-1a [3, 4, 10, 24].CXCL8 / IL-8 is a chemokine responsible for the recruitment of neutrophils and white blood cells. [21, 25] CXCL8 / IL-8 tested in the clinical material did not show a statistically significant increase in the first measurement, although its contribution to the recruitment of neutrophils into the cerebrospinal fluid was shown in animal models(see Table 2) [3, 24]. Moreover, the subsequently tested level of the chemokine CCL3 / MIP-1a, which is responsible for neutrophil recruitment, in the first measurement was lower than in the control group(see Table 2) [21]. At the time of diagnosis, the pathogen was present in the CSF, causing a significant reduction in its glucose level, which was not observed in the control group (see Table 2). This may result from an insufficient influx of leukocytes from the peripheral blood. The major mechanism of virulence of *S*. *epidermidis* is the formation of biofilm, which allows the pathogen to avoid elimination by the central immune system. Overall, the biofilm protects the bacterial strain against antibiotics, biocides, bacteriophages, predatory amoebas and phagocytic white blood cells [3, 5]. The entire structure takes nutrients and minerals from the host’s organism. The biofilm formation by bacterial cells requires synchronized expression of their genes which are responsible for the biosynthesis of polysaccharide intercellular adhesion substance (PIA). Its physicochemical properties play an important role in primary adhesion of the pathogen cells to polymeric materials such as an intraventricular catheter. The PIA produced by *S*. *epidermidis* consists of 1–6 β N-acetylglucosamine. Proliferation of the bacterial cells induces maturation of the biofilm. The consequence of this process is an increase in thickness and density of the biofilm. After reaching its critical mass, the biofilm releases the bacterial cells into the environment [5, 15, 16].

TLR 2 and TLR 9 are present in the biofilm. The immune response to the biofilm differs significantly from the response of the structures of the CNS to the pneumococcal infection, which is described above [15]. Unlike in the case of pneumococcal meningitis, little is known about the innate immune response of the CNS to *S*. *epidermidis* infection. Knowledge of this is based mainly on animal models. TLRs are assumed to play an important role in this process. There are 11 known TLRs in humans [21]. The receptors are located in the choroid plexus and the meninges. TLR-2 is expressed on the outer surface of microglial cells [9, 21].TLR2, being a surface receptor, is particularly involved in the induction of the immune system by S. aureus invasion. This receptor can recognize bacterial peptidoglycans, lipopeptides and lipoproteins [21]. One of the understood immune pathways involves TLR2 present on astrocytes. They induce transformed astrocyte lines to produce pro-inflammatory interleukins such as IL-6, MCP-1 (monocyte chemoattractant protein-1) and IL-8 [5, 24, 26]. The receptors expressed on astrocytes and microglia can be stimulated directly by the pathogen itself and, in addition, by other pro-inflammatory cytokines, such as IL-1β and TNFα [3, 12, 21, 25]. Toll-like receptors 9 are intracellular receptors that recognize the unmethylated cytosine-phosphate-guanine (CpG) motif in the pathogen DNA. They play a role in the recognition of *S*. *aureus* in cases in which it invades the human organism [18]. APCs may be involved in the innate immune response, and, additionally, in the initiation of an inflammatory process. They may be present in the cerebrospinal fluid but not in particularly large numbers. They may also be located in the choroid plexus, the perivascular space and the meninges [10, 12, 20, 26].

At this stage IL-6 seems to play an important role. It is synthesised as a consequence of the exposure of microglia to the pathogen. It can also be secreted by resident macrophages. Moreover, cytokines produced by the microglia can stimulate astrocytes to produce IL-6. This cytokine may stimulate recruitment of peripheral white blood cells. As a result of the secretion of this cytokine, peripheral B lymphocytes can be transformed into plasmocytes. This interleukin is also responsible for the stimulation of haematopoiesis of T lymphocytes and monocytes. T cells attack the CNS earlier than B cells. Microglia and, possibly, astrocytes may be involved in the presentation of pathogen antigens to T lymphocytes [2, 12, 17].The time required to recruit neutrophils ranges from 6 to 24 hours [10–12, 27]. In general, in the first measurement, the white blood cell lines showed a marked increase in counts and percentages in comparison to the control group. Similar results were obtained for the counts and percentages of neutrophils and lymphocytes(see Table 1). Interestingly, negative correlations were found between the percentages of neutrophils and lymphocytes and between the percentages of neutrophils and monocytes in the peripheral blood(see Table 3). This observation may be explained by the fact that neutrophils play an important role in clearing the cerebrospinal fluid from the bacterial strain [10]. The highest count of white blood cells in the peripheral blood in the first measurement can indicate intense interleukin-mediated proliferation and recruitment of neutrophils, since the pathogen was still present in the cerebrospinal fluid(see Table 1). Despite the recruitment of these cell lines in the peripheral blood, the level of pleocytosis in the cerebrospinal fluid did not differ significantly from its level measured in the control group(see Table 2). However, the first measurement showed a positive correlation between the level of pleocytosis in the CSF and the count of neutrophils in the peripheral blood(see Table 3). Thus, a delay in the entry of white blood cells into the cerebrospinal fluid was observed. This could be explained by the fact that the influx of neutrophils into the cerebrospinal fluid requires breaking the BBB. This happens as a result of the secretion of cytokines [10, 12]. Chemoattraction of the neutrophils also requires synthesis of chemokines. The delayed increase in the level of cerebrospinal fluid pleocytosis can be explained by a delayed disruption of the BBB, since in the case of direct introduction of the pathogen into the ventricular system the induction of the peripheral immune system begins later than in the case when the pathogen enters the CNS by the conventional route. Local cytokine secretion activates the serine-threonine protein kinase 2 (NF-κB) and increases production of CSF in the choroid plexus **[**9, 10, 12, 18, 27, 28**].** IL-6 is classified as a pro-inflammatory cytokine, but its role is more complicated. It may also act as an anti-inflammatory lymphokine. Analyzing the pattern of white blood cells and the correlation between the neutrophil chemokines examined in the study (CXCL8 / IL8, CCL3/MIP-1a), it seems that in the study group IL6 acts as cytokines increasing the permeability of the BBB. Elevated levels of cytokines occur not only in an inflammatory process but may also accompany damage caused by increased intracranial pressure resulting from a ventriculo-peritoneal valve dysfunction (DAMP) [2, 3, 9, 10, 19, 29]. This may explain why the level of pleocytosis correlates positively with the level of IL 6. The complex role of IL-6 is illustrated by significantly negative correlations between its level and the levels of glucose, CXCL8/IL-8 and CCL3/MIP-1a. Moreover, positive correlations between the levels of glucose and CCL3 / MIP-1a as well as between the levels of glucose and CXCL8 / IL8 were revealed(see Table 5). Considering the glucose level as a marker for the clearance of the pathogen strain from the cerebrospinal fluid, the anti-inflammatory effect of IL-6 can be explained by the negative correlations with the parameters mentioned above [5, 24, 28]. Analyzing the parameters of the cerebrospinal fluid collected from infected patients leads to the conclusion that, despite the induction of the cellular response in the peripheral blood, it was the humoral response that was more visible. Despite the statistically significant relationship between the levels of pleocytosis and protein, the highest protein level detected in the first measurement was more pronounced than the increase in the level of pleocytosis. The protein level was higher than in the control group(see Table 5). Complement components may be produced not only by the liver, but also by peripherally circulating macrophages, monocytes, lung and gastrointestinal endothelial cells. Locally, neurons and astrocytes, stimulated by pro-inflammatory cytokines, may produce complement components such as C3. Moreover, microglia can stimulate production of C1q, C3, C4, C5a by the peripheral blood cells. C5a is responsible for the increase in the levels of pleocytosis and protein in CSF. Complement component C1q can be produced in the central nervous system, not only by nervous structures but also by microvascular pericytes [2, 30, 31].Animal models in which the humoral immune response was generated intrathecally have been described. The intrathecal immunoglobulin production can be clinically measured using Reibergram (the ratio of the CSF immunoglobulin to the serum immunoglobulin) [2, 10, 30, 31]. The Reibergram may be an indicator of proper functioning of the blood-CSF barrier(BCB) [30, 31]. A more compelling argument is that one of the functions of IL6 is stimulation of proliferation of B cells, and, consequently, the increase of their count in the peripheral blood. Subsequently, IL-6 stimulates conversion of B lymphocytes into plasmocytes, which produce antibodies in a great quantity [18]. However,the most probable is that, the early increase in the protein level in the cerebrospinal fluid of infected patients may be explained by the fact that albumins cross the BBB earlier than neutrophils. The immunoglobulins enter the CSF mainly by the process of receptor-mediated transendocytosis [4, 22, 23].

The second measurement showed the situation in which the cerebrospinal fluid had been cleared of the bacterial strain, but the immune response was still not extinguished. The inflammatory process in the central nervous system is additionally modified by other chemoattractant substances produced by peripheral macrophages, neutrophils and endothelial cells [10, 24]. Pro-inflammatory IL-6 and TNFα are produced by macrophages, monocytes and leukocytes present in the peripheral blood [12]. The most important pro-inflammatory cytokine is TNFα. It is responsible for the influx of polymorphic neutrophils within 6 to 24 hours after detection of the pathogen. It is also responsible for disruption of the BBB. Chemokines induce the influx of neutrophils into the central nervous system [4, 22, 32]. Neutrophils attack the central nervous system when the BBB is broken down and they are recruited by chemokines [16, 23]. An additional mechanism responsible for increasing the BBB permeability is neurogenic stimulation. It has been shown that the permeability may depend on activation of the β2-adrenoreceptors [11]. The path of the pathogen invasion leads through the cerebral venous system. Leukocytes can cross the BBB via the transcellular route or the paracellular one [16, 23]. BBB permeability depends on matrix metalloproteinases (MMPs), which are Zn^+ 2^ and Ca^+ 2^ dependent endopeptidases. They are produced mainly by neutrophils or, to a lesser extent, by macrophages. TNF can also stimulate their production by endothelial cells. They are responsible for the breakdown of laminin, fibronectin, proteoglycans and type IV collagen in the extracellular matrix [10]. After the BBB is disrupted, peripheral leukocytes may enter the perivascular space of the brain and, subsequently, the cerebrospinal fluid [16, 23]. The leukocytes bind to pneumococci with the complement in the process of immunoglobulin-assisted opsonization. Neutrophils destroy pneumococci by phagocytosis. After the pneumococci get enclosed in endosomes, which form and join the lysosomes, they are killed by nitrogen oxides, and, additionally, by lysosomal enzymes. In the extracellular matrix the pneumococci are neutralized by nitrogen oxides released to the outside by the neutrophils. Unfortunately, the destruction is not limited to the pathogen, but also affects other neural structures [10]. Neutrophils cannot regenerate their inflammasomes. They live shorter than monocytes. Their life expectancy ranges from 3 to 135 hours [11]. The population of white blood cells that flock across the BBB consists mainly of granulocytes, neutrophils and, to a lesser extent, monocytes (10%) [3, 10, 24].Despite lack of detectable pathogens in the microbiological test of the cerebrospinal fluid, the glucose level did not differ significantly from the one in the first measurement(see Table 2). The neonatal blood glucose to cerebrospinal fluid glucose ratio was less than 0.6. In babies over 2 months old, the same quotient was lower than 0.4 [33]. The hypoglycorrhachia is usually caused by the presence of a persistent pathogen in the cerebrospinal fluid [34]. Non-infectious causes of low CSF glucose level reported in literature are ischemic / haemorrhagic stroke, neoplasmic processes, lymphoma / leukaemia and neurosarcoidosis [34]. The most probable cause of hypoglycorrhachia in this study may be persistent aseptic meningitis, as a consequence of the active immune response. In clinical trials performed in 1976 on a group of 181 children with hypoglycorrhachia, whose age ranged from 1 week to 14 years, in 8% of the children the hypoglycorrhachia was caused by aseptic meningitis [34]. Thus, increased glycolysis may result from increased metabolism of bacterial strains or cells of the immune system that are overrepresented in the cerebrospinal fluid [34]. All the specimens obtained from the children in the study group and in the control group were free from pathogens causing hypoglycorrachia(see Table 2). A noticeable decrease in the counts and percentages of peripheral white cells in almost all analyzed lines was observed. The only exception was the percentage of monocytes. This parameter showed no significant difference between its values in the second and the first measurements(see Table 1). In the second measurement there were statistically significant correlations between the level of IL-6 and the count of monocytes, as well as between the level of IL-6 and the percentage of monocytes. This correlation was expected, since IL-6 acts as a chemoattractant for monocytes (see Table 3) [5, 24, 26]. In the second measurement the deepest recorded decrease in the value of a white blood cells parameter concerned the percentage of lymphocytes. It was lower than in the control group. This observed large reduction could be caused by the influx of the cell lines mentioned above into the cerebrospinal fluid(see Table 1). This is supported by the fact that the level of CSF pleocytosis was the highest in the second measurement(see Table 2). The aforementioned dependence can illustrate the extinguishment of the humoral response to the invasion of the pathogen. This may be explained by the fact that the bacterial strain has been removed from the cerebrospinal fluid by intrathecal administration of an antibiotic. The peripheral immune system is involved not only in the recruitment of leukocytes into the central nervous system, but also in the humoral response. In response to an invasion of pathogens into the central nervous system, the liver begins to produce acute phase proteins. They also contain components of the complement system [10, 24]. The complement system induces immunoglobulins and helps phagocytes to eliminate pathogens. This is a part of the humoral response, similar to the immunoglobulins (IgA, IgB, and IgMB) produced by lymphocytes. Both factors of the humoral immune response reach the cerebrospinal fluid penetrating across BBB by receptor-mediated trans-endocytosis [23, 30, 31, 35]. It is possible that some elements of the humoral response are synthesized intrathecally [2, 23, 30, 31]. Immunoglobulins cooperate with neutrophils to clear the cerebrospinal fluid from the pathogens. An interesting issue is the reason for the discrepancy between the levels of protein and pleocytosis in the examined material(see Table 2). One of its possible explanations is the revealed large decrease in the count of peripheral blood lymphocytes(see Table 1). This may be due to the fact that in the case of a ventriculoperitoneal valve infection the main source of immunoglobulin are peripheral plasmocytes. Moreover, in the second measurement, a negative correlation between the levels of CSF glucose and protein was found(see Table 5). Perhaps this may be related to the removal of the bacterial strain and less albumin penetration through the damaged BBB. Negative correlations between the count of lymphocytes in the peripheral blood and the level of protein in the cerebrospinal fluid and between the percentage of lymphocytes in the peripheral blood and the level of protein in the cerebrospinal fluid were recorded(see Table 3). However, there was still a positive correlation between the CSF levels of IL6 and protein(see Table 5). In the analyzed clinical material, sterilization with intraventricular administration of an antibiotic (vancomycin) took an average of 20.2 days. For all the children in the study group the route of administration of the antibiotic was selected taking into account the need to bypass the BBB and the BCB. This allowed a therapeutic level of vancomycin to be obtained and antibiotic resistance caused by the bacterial biofilm to be avoided [1, 5, 6, 8, 14, 36, 37]. The highest level of the CSF pleocytosis was detected in the second measurement. Moreover, in the second measurement all the levels of the analyzed cytokines in the CSF also reached their highest values. This could have caused the influx of peripheral blood cells into the cerebrospinal fluid. Some of them were also able to produce cytokines(see Table 2). Thus, in the second measurement the level of cytokines in the cerebrospinal fluid may have increased due to the influx of the peripheral blood cells into it. The cells also produced pro-inflammatory interleukins. This is shown by statistically significant positive correlations between the levels of the studied chemokines (CXCL8 / IL8, CCL3 / MIP-1a) and the level of pleocytosis(see Table 5). Although the level of CXCL8 / IL8 was surprisingly high, it did not exceed the level of this interleukin in the control group. First of all, the explanation for the significantly higher level of CXCL8 / IL8 in the control group is the immune response to damage of the ependymal lining, which is caused by an increase in intracranial pressure as a consequence of hydrocephalus(see Table 2) [2, 3, 9, 10, 19]. Another possible explanation is that CXCL8 / IL8 did not play an important role in the immune response to ventriculo-peritoneal valve infections. However, this is less likely due to the aforesaid positive correlation between the levels of CXCL8 / IL8 and pleocytosis in the CSF(see Table 5). It seems that IL-6 stimulates secretion of the studied chemokines, which explains the statistically significant correlation between the levels of CCL3/

MIP-1a and pleocytosis. The highest level of chemokines and cytokines in the second measurement indicates an impairment of the BBB(see Table 2). The severe inflammatory reaction may be the reason for infection-induced neuroapoptosis [3, 10, 28, 29]. The immune response can lead to apoptosis of nerve cells. Therefore, it is important to discontinue this process once the bacterial strain has been removed from the CNS. The apoptosis can be induced by oxidative stress occurring in the immune response or be a consequence of immune-mediated coagulopathy [3, 10]. Sources of the oxidative stress can be both peripheral neutrophils attacking the central nervous system and local immune structures of the central nervous system. As a result of the oxidative stress, nitric oxide can be formed. Nitric oxide leads to a disturbance in the BBB and stimulates pro-inflammatory cytokines. Superoxide anion O2-, hydrogen peroxide H2O2 and hydroxyl radicals make up reactive oxide species. NO (nitrogen oxide) with H2O2 (hydrogen peroxide) can form peroxynitrite (ONOO-), which is a highly oxidizin compound. Peroxynitrite can damage the CNS lipid peroxidation and DNA fragmentation as a consequenece of the activation of poly (ADB-ribose)polymerase (PARP). Coagulopathy, which is uncontrolled coagulation, together with fibrinolysis lead to nervous cell apoptosis, as it has been demonstrated in the model of pneumococcal meningitis. Local strokes or haemorrhages can result from a number of reasons, such as oedema of a microvascular wall, infiltration of a microvascular wall or intravascular coagulation [10]. The changes in the CSF can also result from aseptic inflammation of the ventricles [34].

The third measurement was performed at the moment when the cerebrospinal fluid was free from bacterial strains but the immune response still seemed to exist. The immune response includes not only induction of an inflammation but also its inhibition. Anti-inflammatory cytokines play a role in extinguishing of the entire process. This is particularly important after the bacterial strain has been removed from the cerebrospinal fluid. The anti-inflammatory cytokines known to be involved in pneumococcal meningitis are IL-10 and TGFβ. IL-10 is responsible for the downregulation of pro-inflammatory cytokines and co-stimulatory molecules produced by macrophages. IL-10 causes an impairment of neutrophil phagocytic and their killing capacities [2– 4, 9, 10, 12, 21, 22]. The anti-inflammatory immune response stops inflammation and takes part in the repair of nerve structures. IL10 and its anti-inflammatory agonists inhibit TNFα [4]. IL-6 has both anti-inflammatory and pro-inflammatory effects[10, 12, 22]. The elimination of the pathogen was clearly visible, because in the third measurement the glucose level was the highest of all the results in the study group(see Table 2). The count of white blood cells and the count and percentage of neutrophils in the peripheral blood were significantly lower than in the first measurement, performed in the initial phase of the immune response(see Table 1). The count of neutrophils was significantly negatively correlated with the level of glucose(see Table 3). This may explain the hypoglycorrhachia in the second measurement. Interestingly, there was still an increase in the counts and percentages of lymphocytes and monocytes(see Table 1). They were higher than in the control group. Moreover, a positive correlation was found between the counts of monocytes and lymphocytes(see Table 4). These are the cell lines responsible for the humoral response. The function of the cell lines was illustrated by a significantly positive correlation between the count of lymphocytes and the protein level. On the other hand, the correlation between the percentages of lymphocytes and neutrophils was negative(see Table 4). Surprisingly, contrary to the cellular response, in the third measurement the tested cytokine levels dropped to the values obtained in the control group or even below them. The higher counts and percentages of monocytes and lymphocytes than those in the control group as well as the count of lymphocytes and the higher percentages of monocytes and lymphocytes than in the second measurement could be explained by the fact that they secrete anti-inflammatory cytokines(see Table 1) [4]. Recovery from the bacterial infection showed a negative correlation between the levels of the analyzed chemokines and glucose(see Table 5). The protein level was negatively correlated with the glucose level, which is considered to be an indicator of the clearance of cerebrospinal fluid from pathogen strains(see Table 5). Nerve stem cells are involved in the protection of the central nervous system from inflammation. They inhibit conversion of Th1 lymphocytes into Th2 lymphocytes [20]. Another anti-inflammatory cytokine, TGF β, plays an important role in inhibition of maturation of regulatory T cells (Tregs). It also inhibits differentiation and maturation of TH17and hinders transformation and maturation of T1 and T2. Another type of anti-inflammatory properties of some cytokines is their ability to inhibit the production of pro-IL-6 and IL-1β inflammatory cytokines by macrophages. Some cytokines may also inhibit production of TNF in the microglia [3, 10, 24]. The immune response of peripheral white blood cells depends on the balance between the pro and anti-inflammatory processes [32].

## Conclusion

1. Infection of a ventriculoperitoneal shunt caused by *S*. *epidermidis* evokes a very early peripheral blood response. This results in the highest levels of neutrophils, lymphocytes, and monocytes in infected patients prior to the treatment.
2. In children affected by a ventriculoperitoneal valve infection, the protein increased level detected in the cerebrospinal fluid precedes the increase in the level of pleocytosis. The humoral immune response in the cerebrospinal fluid may result from secretion of immunoglobulins by the peripheral blood lymphocytes, which takes place before the invasion of the inflammatory cell lines into the cerebrospinal fluid through the impaired BBB.
3. The increased level of pleocytosis in the cerebrospinal fluid may result from recruitment of the peripheral white blood cells to the cerebrospinal fluid and it may explain the significant reduction in the counts of neutrophils, lymphocytes, and monocytes in the peripheral blood after sterilization of the cerebrospinal fluid.
4. The highest level of cytokines in the cerebrospinal fluid is achieved when the pathogens are cleared as a consequence of secretive activity of the local immune system of the CNS and peripheral immune cells interacting together.
5. Phagocytes, and, in particular, monocytes, play an important role in the normalization of the cerebrospinal fluid parameters after the elimination of *S*. *epidermidis*. The normalization of the levels of glucose, protein and pleocytosis in the cerebrospinal fluid coincides with the highest count of peripheral blood monocytes.
6. The only parameter that can indicate that the local immune response of the central nervous system plays an important role in extinguishment of the inflammatory process is the statistically proved correlation between the level of IL-6 in the cerebrospinal fluid and the count of monocytes in the peripheral blood in the third measurement.
7. The compound role of IL -6 in initiation and extinguishment of the inflammatory process illustrates statistically positive correlation between that cytokine and CSF pleocytosis in the beginning of and recovery from infection. The rest of analysed chemoattractant cytokines such as CXCL8 / IL-8, CCL3 / MIP-1a act as proinflammatory factors, which illustrates statistically significant correlation between them and CSF pleocytosis in the second measurement

## Supporting information

S1 Data (XLS)

S2 Data (XLS)

S3 Data (XLS)

## Abbreviations

APCs: antigen presenting cells
BBB: blood-brain barrier
BCB: blood-cerebrospinal fluid barrier
CNS: central nervous system
CSF: cerebrospinal fluid
WBC: white blood cell counts
